# Anti-*Plasmodium* Activity of Angiotensin II and Related Synthetic Peptides

**DOI:** 10.1371/journal.pone.0003296

**Published:** 2008-09-29

**Authors:** Ceres Maciel, Vani Xavier de Oliveira Junior, Marcos Antonio Fázio, Rafael Nacif-Pimenta, Antonio Miranda, Paulo F. P. Pimenta, Margareth Lara Capurro

**Affiliations:** 1 Department of Parasitology, Institute of Biomedical Sciences, University of São Paulo, São Paulo, Brazil; 2 Natural and Human Science Center, Federal University of ABC, Santo André, Brazil; 3 Department of Biophysics, Federal University of São Paulo, São Paulo, Brazil; 4 Laboratory of Medical Entomology, René Rachou Institute of Research, Oswaldo Cruz Foundation–FIOCRUZ, Belo Horizonte, Brazil; Columbia University, United States of America

## Abstract

*Plasmodium* species are the causative agents of malaria, the most devastating insect-borne parasite of human populations. Finding and developing new drugs for malaria treatment and prevention is the goal of much research. Angiotensins I and II (ang I and ang II) and six synthetic related peptides designated Vaniceres 1-6 (VC1-VC6) were assayed *in vivo* and *in vitro* for their effects on the development of the avian parasite, *Plasmodium gallinaceum.* Ang II and VC5 injected into the thoraces of the insects reduced mean intensities of infection in the mosquito salivary glands by 88% and 76%, respectively. Although the mechanism(s) of action is not completely understood, we have demonstrated that these peptides disrupt selectively the *P.gallinaceum* cell membrane. Additionally, incubation *in vitro* of sporozoites with VC5 reduced the infectivity of the parasites to their vertebrate host. VC5 has no observable agonist effects on vertebrates, and this makes it a promising drug for malaria prevention and chemotherapy.

## Introduction

Malaria remains one of the world's most deadly diseases. However, efforts to control this disease are hampered by drug resistance in parasites, insecticide resistance in mosquitoes, and the lack of an effective vaccine [1]. Malaria parasites (*Plasmodium sp*) have an intricate and complex life cycle in vertebrate (intermediate) and invertebrate (definitive) hosts, and the multiple developmental forms of *Plasmodium* species are potential targets of distinct antiparasite molecules. For example, the mosquito-stage parasites can be disrupted by natural and engineered peptides, such as the antimicrobial peptides (AMP) cecropin, magainin, defensin, scorpine and cecropin-like peptides [2]–[6]. However, all the AMPs tested thus far fall short of the requirements for an effective anti-plasmodial molecule since they require high concentrations both *in vivo* and *in vitro* to be effective. These results support the search for alternative molecules, including novel peptides with lower effective doses.

Antimicrobial peptides kill bacteria by interfering with metabolism, targeting cytoplasmic components and disrupting membranes [7]. Lipid composition of the target cell membrane is a determining factor in the activity and selectively of AMPs for avoiding damage to host cells [8], [9]. Peptides with parasiticidal effects on sporozoites could be utilized to target parasites within their vector mosquitoes. These same peptides, being harmless to vertebrate hosts, would have a broader application in malaria prophylaxis by targeting circulating sporozoites and preventing the establishment of infection. With this aim, we used the avian malaria parasite, *Plasmodium gallinaceum*, as a model system to search for drugs with both properties: toxicity to parasites and harmless to vertebrates and invertebrate hosts. We report here that a synthetic angiotensin II-related, vertebrate-inactive peptide (VC5) kills *P. gallinaceum* sporozoites *in vitro* and *in vivo* and discuss possible applications of this finding in the development of novel malaria control strategies.

## Results

### Ang I, ang II and VC1-VC6 peptides reduce the number of P. gallinaceum sporozoites in salivary glands

**Figure 1:**
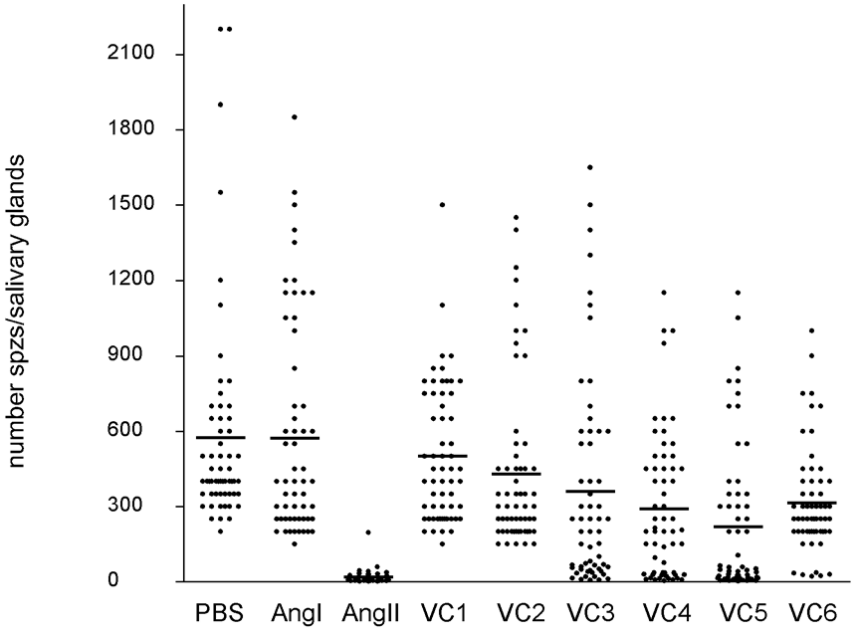
Numbers of sporozoites in salivary glands of angiotensin I-, angiotensin II- or synthetic peptide-treated *Ae. aegypti.* At day 7 post-infection 0.5 μl of ang I (60 μM), ang II (60 μM), VC1 (60 μM), VC2 (60 μM), VC3 (60 μM), VC4 (60 μM), VC5 (60 μM), VC6 (60 μM) and PBS (control) were microinjected intrathoracically on anesthetized *Ae.aegypti*. Salivary glands were dissected 24 hours after the microinjection and sporozoites counted. Kruskal–Wallis tests indicated significant effects (*p*<0.0001 and *p*<0.001) of the peptides in the number of salivary glands sporozoites for ang II and VC5, respectively. VC1, VC2, VC3, VC4, VC6 did not significant effect (*p*>0.05).

Injection of ang II (5–60 μM) in the hemolymph of *P.gallinaceum* infected *Ae. aegypti* reduces sporozoites accumulation in the salivary glands and does not affect mosquito survival (Supporting Materials, Figure S1 and Figure S2). Following this initial observation, synthetic ang II related peptides were designed and tested for their anti-parasite activities. Injection of 0.5 μl of synthetic peptides VC1-VC 6, ang I and ang II (at 60 μM concentration) in infected mosquitoes resulted in reduced numbers of sporozoites in their salivary glands when compared with controls injected with either PBS or ang I (Figure 1). VC1 showed the least effect on accumulation of sporozoites, while ang II and VC5 where the most effective reducing by 88% (*p*<0.0001) and 76% (*p*<0.001), respectively, the number of parasites compared to control infected mosquitoes. Additionally, each peptide was tested for its anti-sporozoite activity in three independent experiments and only ang II and VC5 were consistently effective (*p*<0.005) in reducing the number of salivary glands parasites.

### Ang I, Ang II and VC1-VC6 increase sporozoite membrane permeability

**Figure 2:**
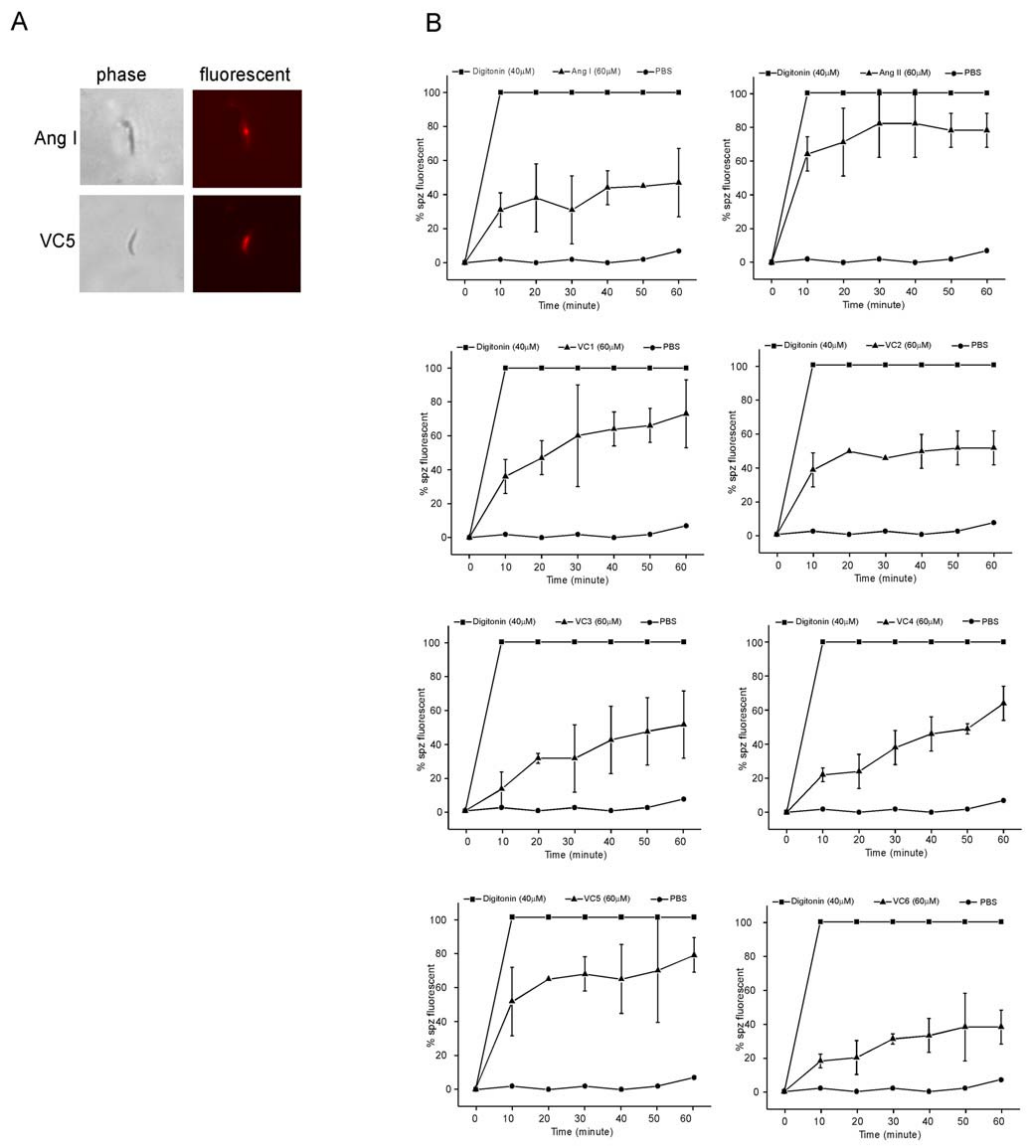
Effects of peptides on membrane permeability of mature sporozoites were incubated with digitonin, PBS, 60 μM of ang I, ang II, VC1, VC2, VC3, VC4, VC5 or VC6. After defined intervals at room temperature, propidium iodide was added and the sporozoites were examined by fluorescent microscopy. A, sporozoites in phase (left) and fluorescent (right) microscopy; B, kinetic curves of membrane fluorescence. Results are the mean of three independent experiments, and bars represent standard errors of the mean. The data were statistical analyzed (60 min) using Fisher's test showing that ang II, VC1 and VC5 are significant different (*p*<0.02) when compared with control. On the other hand, ang I, VC2, VC3, VC4 and VC6 are not significantly different (*p*>0.05).

Mature sporozoites were recovered from salivary glands and incubated *in vitro* with ang II or VC peptides. Cell membrane integrity was monitored at 10 minutes intervals by adding propidium iodide to the treated parasite samples (Figure 2). Sixty-four percent of the sporozoites showed nuclear fluorescence indicative of cell damage after 10 minutes incubation with 60 μM ang II, and 78% showed fluorescence after 30–60 minutes. VC1, VC2, VC3, VC4 and VC6, respectively, resulted in 73%, 51%, 51%, 64% and 38%, sporozoites with fluorescent nuclei after 1 hour incubation. VC5 resulted in a kinetic profile similar to that obtained with ang II. Incubations with 60 μM VC5 yielded 51%, 67% and 78% fluorescent sporozoites at 10, 20 and 60 minutes, respectively. Fluorescence accumulation in the nuclei was observed in only 47% of the sporozoites treated with 60 μM ang I after 1 hour incubation.

**Figure 3:**
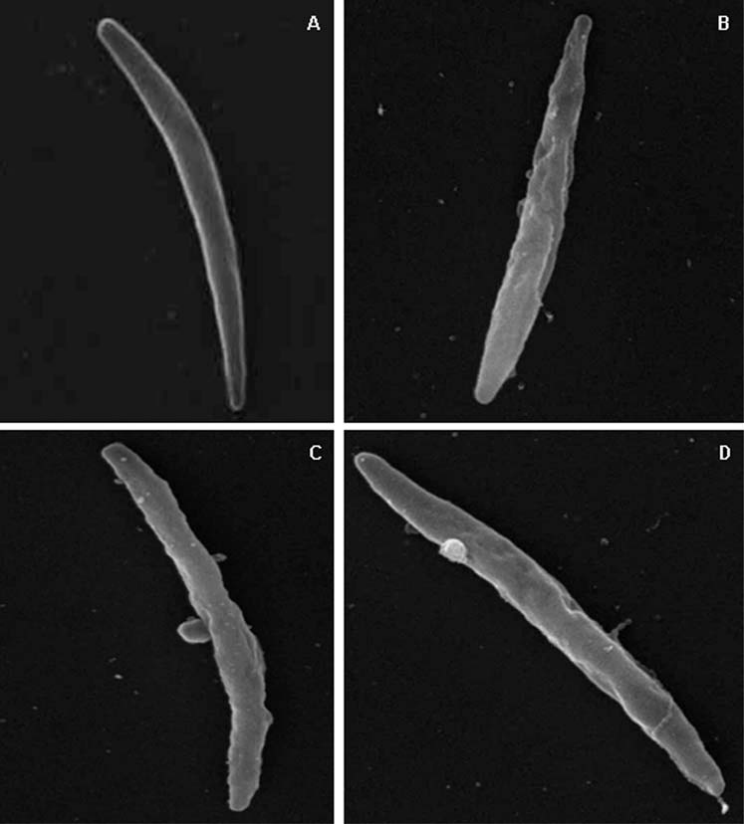
Scanning electron microscopy of salivary gland-derived *P. gallinaceum* sporozoites. Parasites were incubated with PBS (A, control), angiotesin II (B) and VC5 (C and D). Note the smooth surface of the non-treated sporozoites (PBS-control) with the rough surface of the drug-treated parasites (ANG II and VC5).

Further evidence of the association of ang II and VC5 peptides with the sporozoite cell membrane was obtained by scanning electron microscopy (Figure 3). Sporozoites incubated with VC5 show surface damage evidenced by cytoplasmic protrusions, and this is consistent with the propidium iodide staining of their nuclei (Figure 2).

### Parasitological evidence of inactivation of salivary gland sporozoites by VC5

Vertebrate-infective parasites harvested from mosquito salivary glands were incubated *in vitro* with VC5 and then inoculated into chickens. Both the infection rates (15%) and the pre-patent periods (7–21 days) were affected (Table 1). Only four of nine chickens challenged with VC5-treated sporozoites became infected, and those showed an increased pre-patent period, 7 to 21 days, compared to six days post-infection in the control group. Parallel incubations *in vitro*, without VC5, under identical conditions did not diminish the infectivity of parasites, as demonstrated by the infection of 100% of chickens challenged with these control samples. This preliminary experiment further support that VC5 interacts with malaria parasites cell membrane indicates that this interaction results in non-infectious sporozoites.

**Table 1:** Infectivity for chicks of *P. gallinaceum* spzs from salivary glands after incubation with 60 μM of VC5.

	Inoculum size^a^	Chicks n° infected/n° injected	Prepatent period^b^ (Days)
**VC5 (60** μ**M)**	50	1/3	9
	500	3/5	7, 12, 21
**PBS**	50	5/5	6
	500	1/1	6

a The chicks were i.v. inoculated after 1 hour incubation of spzs and VC 5 or PBS.

b Pre-patent period is 6 days.

### Agonistic activities of synthetic VC1-VC6 and ang II

In addition to applications of VC5 and ang II to target sporozoites in the vector mosquitoes, these peptides also could be useful to block parasite development in vertebrate hosts. Angiotensin II is a well-known direct activator of smooth muscle in the vasculature, constricting arteries and veins and increasing blood pressure. Assays of ileum contraction, commonly used to determine vasoconstriction activities, were utilized to test agonistic activities of the peptides on guinea pig (GPI), chicken ileum (CI) and rat uterus (RU) preparations. When compared with ang II (100% activity), VC1 had 5%, 0% and 2.0% of agonist activity on GPI, CI and RU, respectively (Table 2). VC2 had 10%, 0% and 2.9% of activity for GPI, CI and RU. VC3, VC4, VC5 and VC6 activities were less then 0.3%, consistent with the interpretation that the VC1-6 peptides have low or no agonistic action.

**Table 2:** Effects of peptides on vertebrate tissues

Peptide	Compound Name^a^	HPLC^b^	CZE^c^	MS^d^		Agonist Activity^e^				Hemolytic Activity
				Calculated	Measured	Guinea Pig Ileum	Rat Uterus	Rat blood pressure	Chicken Ileum	
Ang II	—	99	98	1046-2	1046.5	100	100	100	100	neg
VC 1	Cyclo(0-1a)[Asp^0^, endo-(Lys^1a^)]-All	97	97	1271.5	1272	5.0	2.0	2.0	0.0	neg
VC 2	[Asp^0^, endo-(Lys^1a^)]-All	95	96	1289.5	1289	10.0	2.9	2.1	0.0	neg
VC 3	Cyclo(1a-2a)[endo-(Asp^1a^), endo-(Lys^2a^)]-All	95	95	1271.5	1272	0.2	0.3	0.4	0.0	neg
VC 4	[endo-(Asp^1a^), endo-(Lys^2a^)]-All	98	95	1289.5	1291	0.0	<0.1	0.5	0.0	neg
VC 5	Cyclo(2a-3a)[endo-(Asp^2a^), endo-(Lys^3a^)]-All	95	95	1271.5	1272	0.0	<0.1	<0.1	0.0	neg
VC 6	[endo-(Asp^2a^), endo-(Lys^3a^)]-All	97	96	1289.5	1289	0.0	0.2	<0.1	0.0	neg

a In convention with IUPAC-IUB, Biochemistry 1967, 6(1), 362-368.

b Percent purity determined by RP-HPLC using buffer systems: A = 0.1% TFA/H_2_O and B = 60% CH_3_CN/0.1% TFA in H_2_O with a gradient slope of 3% B/min, at flow rate of 1,5 ml/min on a Beckman C_18_ column (4,6×150 mm, 5 μm particle size, 300 Å pore). Determination at λ = 214 nm.

c CZE was done using a Waters System, model CIA (Capillary Ion Analyzer) by hydrostatic injection in 25 seconds, utilizing Phosphate buffer pH 2,50, silica capillary (75 μm ID×60 cm length), field strength of 20 kV at temperature 30°C. Determination at λ = 214 nm.

d Molecular weights were determined by Maldi-TOF using a α-ciano-4-hydroxicinamic acid matrix.

e Biological activities of VC1-6 were compared to those of angiotensin II on the guinea pig ileum, rat uterus, rat blood pressure and chicken ileum. The hemolytic activity of angiotensin II and its analogues was measured at a peptide concentration ranging from 0.19 to 100 μM.

### Stability and absence of hemolytic activity of ang II and VC1-VC6

Hemolytic activity was evaluated using fresh human erythrocytes harvested and incubated with serial dilutions of the peptides, and concentrations varying between 0.19–100 μM. Hemolysis was not detected even at the higher concentrations (Table 2). Ang II and VC1-VC6 analogue stability in blood was evaluated in non-heat inactivated human serum, demonstrating that the peptides were degraded within 30 minutes of incubation, as determined by LC/ESI-MS (data not shown).The quick clearing of the molecules, low or no agonist activity and the absence of hemolysis caused by the peptides are indicative of their general non-toxicity to vertebrates.

## Discussion

A recent worldwide trend of attention to malaria control has come with accompanying financial support directed towards the development of new drugs, anti malarial vaccines and alternatives for vector population management [10]. Technical developments such as the sequencing of the genomes of malaria parasites [11] and the vector mosquito *Anopheles gambiae* [12], and the ability of generating parasite-resistant genetically-modified mosquitoes [13] have been heralded as keystones for the development of novel malaria control tools. Our efforts to develop peptides with anti-malarial activities led us to determine serendipitously that angiotensin II affects negatively the development of malaria parasites. Here we report our initial examination of the mechanisms that result in such effect.

Ang II is derived from the constitutively-produced angiotensinogen, a peptide of the serpin family released into the circulation mainly by the vertebrate liver. When blood pressure decreases in the kidneys the enzyme rennin is produced and cleaves the peptide bond between the leucine (Leu) and the valine (Val) residues creating the ten amino acid peptide angiotensin I (ang I). Ang I has little biological effect and it is processed further to ang II by removal of two of the terminal amino acid residues. Ang II plays an endocrine role in the regulation of blood pressure, fluid and electrolyte homeostasis. In addition, studies have shown that numerous tissues and organs contain their own locally-generated angiotensin products that exhibit tissue-specific activities [14]. Therefore, the biological activities of ang II preclude its utilization as an anti-malarial drug. Modifications of the molecular structure of ang II were then considered, and we designed six related peptides (VC1-VC6) that were further analyzed. VC5 displayed no agonistic effects and killed malaria sporozoites in the conditions utilized in our experiments. The mechanism of parasite killing was further investigated by biochemical and morphological applications. VC5 and ang II kill *P. gallinaceum* sporozoites by disruption of their plasma membrane. Moreover, these peptides have no similar effect upon vertebrate cells. The observed specificity may be explained by the markedly different lipid compositions of *Plasmodium* and vertebrate cells plasma membranes [15]. VC5 is a promising molecule to be tested for malaria prevention and chemotherapy.

### Final Remarks

Our research shows that angiotensin-related peptides affect malaria parasite survival. These peptides were engineered successfully to abolish their agonist functions while retaining parasiticidal activity, opening new research possibilities. Presently, the design, synthesis, and biological evaluation of new analogues are in progress in our laboratories with the ultimate goal of generating further information on the structural requirements for ang II analogues bioactivity and conformation. These studies focus on bridge-length optimization and amino acid chirality that may improve molecular stability against protease presents on plasma and result in longer time of action. Additional research is necessary to further characterize the mechanism of action of these peptides to kill *Plasmodium* parasites and direct future efforts in drug development that can contribute to the control of malaria.

## Materials and Methods

### Peptide synthesis, purification and characterization

Angiotensin II (ang II)-related peptides were synthesized by a solid-phase method using the t-Boc strategy on a chloromethylated resin [16], with substitutions varying from 0.5–0.8 mequiv/g. The following side-chain protected Boc-amino acids were employed from Bachem Inc. (Torrance, CA): Arg(Tos), Asp(OcHex) and (OFm), His(Tos), Lys(Fmoc) and Tyr(2-Cl-Bzl). Ang ll reagents and solvents were of analytical grade and used from freshly opened containers without any further purification. The N^α^-terminal protections were removed with 50% TFA in methylene chloride in the presence of 2% anisole for 20 min. Couplings were done using 2.5-fold excess of 1,3-diisopropylcarbodiimide/N-Hydroxybenzotriazole (DIC/HOBt) in methylene chloride-dimethylformamide (DCM-DMF) (1∶1, v/v). Both steps were monitored by the ninhydrin test [17]. Couplings times were 1–2 h, and when needed, recouplings of 1 h were done using 2.5-fold excess o-benzotriazol-1-yl-N,N,N′,N′-tetramethyluronium tetrafluoroborate (TBTU) in the presence of excess N-N′-diisopropylethylamine (DIEA) in methylene chloride-1-methyl-2-pyrrolidone (DCM-NMP) (1∶1, v/v) [18]. Boc-His (Tos) DCHA (*dicyclohexylammonium salt*) incorporation employed a 1.5-fold excess of TBTU in presence of excess of diisopropylethylamine (DIPEA). Acetylations were performed with 50% acetic anhydride in DMF for 15 min when required. The orthogonal protection of the side chain of the aspartic acid (OFm) and lysine (Fmoc) residues were used in order to allow the lactam bridge formation with the peptide still attached to the resin [19]. After washes with DCM, DMF, the OFm/Fmoc groups were removed by 20% piperidine in DMF. The peptide-resin was cyclized by reaction with 3.0-fold excess of benzotriazol-l-yl-oxy-tris-(dimethylamino)phosphonium hexa-fluorophosphate (BOP) in the presence of excess DIEA in 20% DMSO/NMP. After washing the cyclization was repeated every 20 minutes. The reaction was followed by the Kaiser ninhydrin test [17]. The dry protected peptidyl-resin was exposed to anhydrous hydrogen fluoride (HF) in the presence of 5% anisole and of 5% dimethylsulfide for 75 min at 0°C. HF excess and scavenger were eliminated under high vacuum. The crude peptides were precipitated with anhydrous diethyl ether, separated from ether-soluble by filtration, extracted from the resin with 5% acetic acid in H_2_O and lyophilized. The crude lyophilized peptides were purified in two steps (triethylammonium phosphate (TEAP) pH = 2.25 and 0.1% TFA) by preparative Reversed Phase-High Performance Liquid Chromatographic (RP-HPLC) on a Waters Associates system (Model Prep 4000), using linear gradients (slope 0.33% B/min). Briefly, they were loaded on a Vydac C_18_ (25×250 mm, 15 μm particle size, 300Å pore size) preparative RP-HPLC column at a flow rate of 7.0 ml/min and eluted with TEAP (pH = 2.25)/CH_3_CN), and detection at 220 nm. Selected fractions were collected and converted to the Trifluoroacetic acid (TFA) salt by loading on a preparative column as mentioned above and eluted using a linear gradient (slope 0.33 or %B/min) containing a mixture of solvents A [0.1% TFA/H_2_O] and B [0.1% TFA in CH_3_CN/H_2_O (75:25)] at a flow rate of 7.0 ml/min. Selected fractions containing the purified peptide were pooled and lyophilized [20]. Analytical RP-HPLC was performed on a Waters Associated system using a linear gradient of 5–95% B for 30 min, using buffer system: A = 0.1% TFA/H_2_O and B = 0.1% TFA-60% CH_3_CN/H_2_O, at 1.5 ml/min, on a Beckman C_18_ column (4.6×150 mm, 5 μm particle size, 300Å pore size) at 215 nm. Capillary zone electrophoresis was done by using a Waters System, model CIA (Capillary Ion Analyzer) by hydrostatic injection in 25 seconds, utilizing Phosphate buffer pH 2.50, λ = 214 nm, silica capillary (75 μm×60 cm), voltage 20 kV at 30°C. Amino acids analyses were made by the ninhydrin method using an automatic analyzer Beckman, model System 6300. Peptides were hydrolyzed (1 mg) using 1 ml of HCl 6 M, in the presence of phenol 0.08 ml to 5% in water at 110°C for 72 hours in atmosphere of N_2_. After hydrolysis, the material was vacuum-concentrated and dissolved in 0.2 M sodium citrate, pH 2,2, filtered through Millipore (pore 0,45 μm) before being injected in the analyzer. The molar relationship of the amino acids was established using the concentration unitary of the closest amino acid of the average for all the residues [21]. The peptides were analyzed on a Micromass spectrometer model TofSpec SE using voltage = 20 KV, suppression at 500 AMU, Mode Reflectron (10 KV) and the α-ciano-4-hydroxycinnamic acid solid matrix. The spectrometer was periodically calibrated with angiotensin I and AII. Purified peptides were characterized (Table 2), by RP-HPLC (Reversed-Phase Liquid Chromatography), CE (Capillary Electrophoreses), AAA (Amino acid Analyses) and MS (Mass Spectroscopy). The characterization results showed that the amino acid proportion were in agreement with the expected values by AAA (data not shown), purity in HPLC and CE showed to be above 95%, and had appropriate molecular weights by mass spectroscopy. (Table 2).

### Agonistic VC5 activity to angiotensin II

Peptide agonist bioassays were performed in isolated guinea pig, rat and chicken ilea comparison the synthetic peptides with ang II. Male guinea pigs (weight: 200–250g) and chickens (30–40g) were sacrificed and the terminal portions of the ileum removed and washed several times with Tyrode solution (8 g NaCl; 0.2 g CaCl_2_; 0.1 g MgCl_2_; 0.2 g KCl; 1 g NaHCO_3_; 0.05 g NaHPO_4_ and 1g of glucose per liter of water). After 15 minutes in nutritive solution at room temperature, the ileum was sectioned (4.5 cm) and fixed in the perfusion chamber with 5 ml of aerated Tyrode solution bubbled with a gas mixture of 95% O_2_/5% CO_2_ at 37±0.5°C. Isotonic contractions were registered by a chimograph (Palmer), under a resting load of 1g. VC5 was dissolved in H_2_O (1.0 mg/ml), diluted in 0.9% NaCl and added to the perfusion chamber. After 90 seconds, the preparation was washed with Tyrode solution. The measurements of agonist activity were done in triplicate.

Blood pressure assays were conducted on female rats (250–300 g) anesthetized with a ketamine-xylazine (Ketamine 80 mg/kg and Xylazine 8 mg/kg) solution and maintained by artificial breathing. A catheter was inserted into the carotid artery and used to register the blood pressure with a pressure transducer (Hewlett-Packard, model 1280C-02), amplifier (Hewlett-Packard, model 8805B) and chart recorder (ECB, model RB102). Isotonic solutions of the peptides were injected in the femoral vein using a polyethylene catheter inserted (Table 2).

### Hemolytic activity

Hemolytic activity was evaluated on fresh human erythrocytes washed three times with phosphate-buffered saline (PBS: 10 mM Na_2_HPO_4_, 1.8 mM K_2_-HPO_4_, pH 7.4, containing 140 mM NaCl and 2.7 mM KCl) ) [22]. Serial dilutions of the peptides with concentrations varying between 0.19–100 μM were incubated in Eppendorf tubes with a suspension of 0.4% erythrocytes in PBS. After 1 h at 37°C, the test tubes were centrifuged at 300g for 5 min at 5°C. Fifty microliter aliquots of the supernatants were transferred to 96-well plates and hemolysis was monitored at 405 nm using a microtiter plate reader. Negative and positive controls were prepared in PBS and in PBS supplemented with 0.1% SDS, respectively. Ang ll of the experiments was performed in triplicate.

### Serum stability

Twenty microliters of an aqueous peptide stock solution (10 mg/ml) were added to 1 ml of 25% pooled non-heat inactivated human serum in PBS and incubated at 37°C. Aliquots of 50 μl were added to 5 μl of TFA at time intervals (0, 10, 20, 30, 60 and 120 minutes). The resulting mixtures were kept at 5°C for 10 min and then centrifuged at 300×g for 5 min. Twenty microliters of the supernatants were injected in a LC/ESI-MS equipment and the components separated using a linear gradient of acetonitrile in acidified water (0.1% TFA) at a flow rate of 0.4 ml/min from 5% to 95% B (0.1% TFA/60% CH3CN/H2O) in 30 min. Peptide consumption mentored as an area decrease under the corresponding peak in the chromatogram allowed the evaluation of the stability of the peptide in serum. All the experiments were performed in triplicates.

### Mosquito rearing and maintenance of the parasite life cycle

The RED strain of *Aedes aegypti* is highly susceptible to *P. gallinaceum* [23] and was used in all experiments. Mosquitoes were reared using standard laboratory procedures [24].

An aliquot of frozen chicken blood infected with *P. gallinaceum* strain 8A was obtained from A. Krettli (René Rachou Institute of Research, FIOCRUZ, MG, Brazil). This sample was used to inoculate and establish initial infections in chickens. All subsequent infections of chickens and mosquitoes were accomplished by feeding mosquitoes on chickens.

### Mosquito injection

Each synthetic peptide was injected (0.5 μl of 60 μM) into a group of naturally-infected mosquitoes seven days after a blood meal, at a time when sporozoites were anticipated to be present in the hemolymph. The same volume of PBS was injected into a control group. Injections were performed using a finely-drawn calibrated glass microcapillary tube to deliver the solutions into the thorax. After 24 hours, individual pairs of salivary glands were dissected, homogenized in 10 μl PBS, placed on a hemacytometer and the sporozoites counted using phase-contrast microscopy. This procedure allowed the examination of the whole salivary gland for sporozoites [25]. All fields of the hemacytometer were examined thoroughly in low parasites densities samples and five fields were averaged for high parasite densities samples.

### Inactivation of sporozoite infectivity

*In vitro* experimental treatments were performed with 50–500 sporozoites isolated from salivary glands 13 days after mosquito infection. Parasites were mixed with synthetic peptides to final concentration of 60 μM. After 1-hour incubation at room temperature, the samples were injected intravenously into two-days-old chickens. Blood smears were taken daily from each chicken (6 to 25 days after inoculation), stained with Giemsa after methanol fixation, and examined by light microscopy at magnification of 1000x. The pre-patent period was calculated as number of days between inoculation and detection of circulating parasites in the chickens.

### Effects of Angiotensin I (ang I,) Angiotensin II (ang II) and Vaniceres 1-6 (VC1-6) on salivary gland-derived Plasmodium gallinaceum sporozoites

Three thousand *P. gallinaceum* mature sporozoites were recovered from salivary glands and incubated with 40 μM digitonin, 60 μM ang I, 60 μM ang II, 60 μM VC1-6 or PBS at room temperature. Cell membrane integrity was then monitored at 10 minutes intervals by adding propidium iodide to the treated parasites.

### Scanning electron microscopy

Freshly-purified sporozoites from salivary glands were incubated in 50 μl PBS at room temperature in the absence or presence of 60 μM ang II and 60 μM VC5. After incubation, 10 μl aliquots were separated and fixed overnight with 2.5% glutaraldehyde in 0.1 M cacodylate buffer, pH 7.2. The glutaraldehyde-fixed samples were rinsed three times in PBS and post-fixed in 1% osmium tetroxide solution in 0.8% potassium ferrycianide in 0.1 M caccodylate buffer pH 7.2 for two hours, and then washed three times in PBS. The samples were dehydrated in ethanol, dried in critical point device with CO_2_ method, mounted in special stubs, coated with platinum particles and analyzed by SEM (JEOL JSM 5600).

### Toxicity assays of VC5 in chicken

The toxic effect of VC5 was evaluated in chickens. Six animals per cage weighing 30–40 g each were housed in groups of 10 in our institutional animal care facility and allowed to adapt for seven days prior to the onset of experiments. Animals were maintained in a temperature-controlled environment (21±2°C), with free access to water and food. All experiments were carried out in accordance with the guidelines of the Institutional Ethics Review Committee (Colégio Brasileiro de Experimentação Animal–COBEA) and Animal Care of the Institute of Biomedical Sciences (Comissão de ética em experimentação animal–CEEA)–University of São Paulo, protocol #133.

### Statistical analysis

The Kruskal-Wallis test, Fisher's Exact test, chi-square test and Mann-Whitney U test (GraphPad InStat version 3.00 for Windows 95, GraphPad Software, San Diego California USA, www.graphpad.com) was used to assess the statistical significance of the differences between control and peptides injected groups.

## Supporting Information

Figure S1 Survival of mosquitoes injected with different ang II concentrations. Ae.aegypti mosquitoes (10 females) were injected intrathoracically (0.5 μl) of ang II at 10 μM, 20 μM, 50 μM, 60 μM, 100 μM and 200 μM. After 24 hour the survival of mosquitoes was scored. Chi-square test indicated that no significant effects of the ang II (*p*>0.5) in mosquito survival for all the experiments. Results are the mean of three independent experiments (20 mosquitoes/group), and bars represent standard errors of the mean.
(0.18 MB TIF)

Figure S2 Numbers of sporozoites in salivary glands of angiotensin I-, angiotensin II- or synthetic peptide-treated Ae. aegypti. At day 7 post-infection 0.5 μl of ang II at 5 μM, 30 μM, 40 μM, 50 μM, 60 μM or PBS (control) were injected intrathoracically in anesthetized *Ae.aegypti*. Salivary glands were dissected 24 hours after the microinjection and sporozoites counted. Mann-Whitney tests indicated significant effects (*p*<0.0001) of the peptides in the number of salivary glands sporozoites at 60 μM concentration.
(0.48 MB TIF)
