# Supplementary figures and images for: Anti-Plasmodium Activity of Angiotensin II and Related Synthetic Peptides
Source: PLoS One. 2008 Sep 29;3(9):e3296. doi: 10.1371/journal.pone.0003296 (PMC2546444; doi:10.1371/journal.pone.0003296)

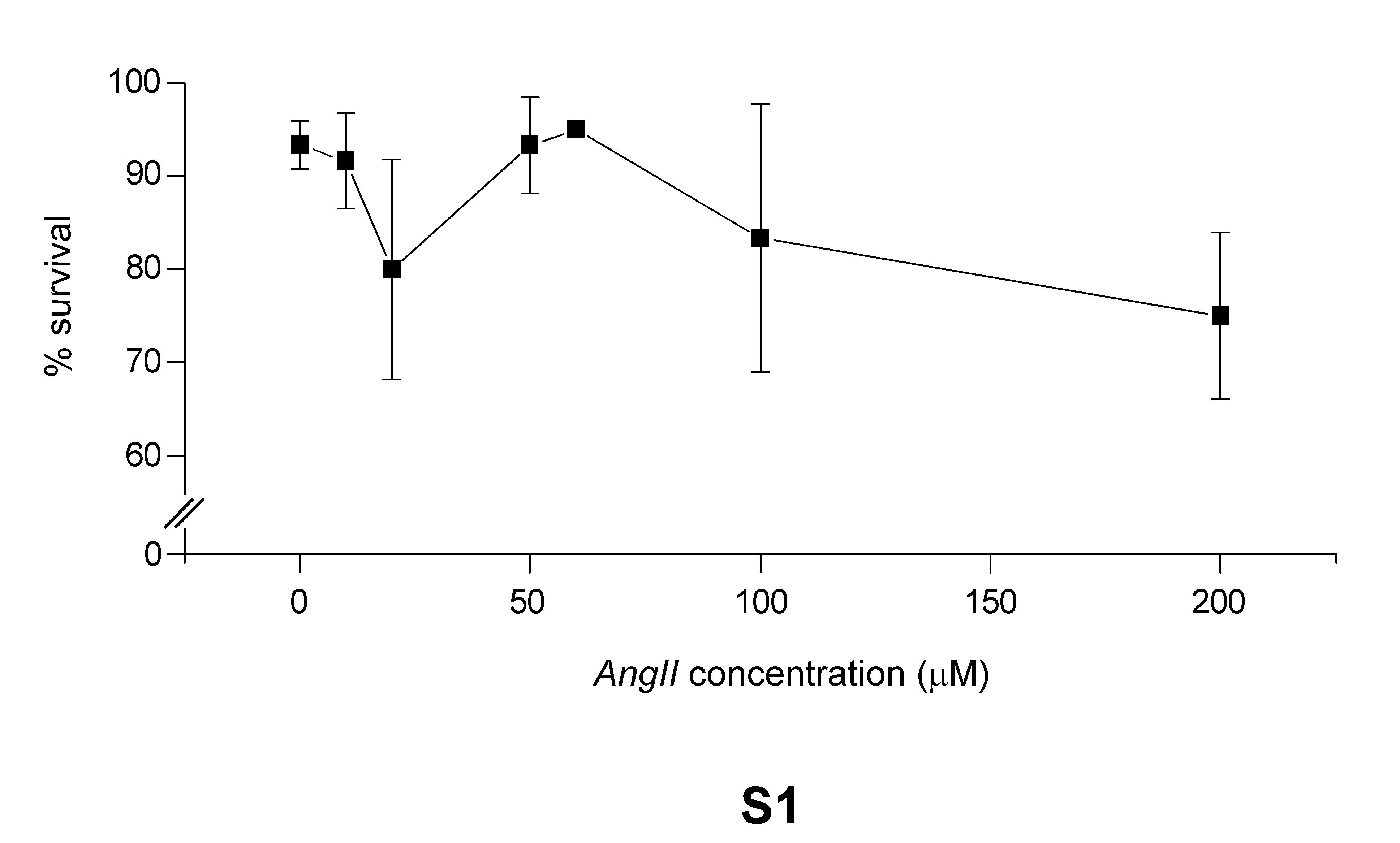

Supplement: Figure S1 — Survival of mosquitoes injected with different ang II concentrations. Ae.aegypti mosquitoes (10 females) were injected intrathoracically (0.5 μl) of ang II at 10 μM, 20 μM, 50 μM, 60 μM, 100 μM and 200 μM. After 24 hour the survival of mosquitoes was scored. Chi-square test indicated that no significant effects of the ang II (p>0.5) in mosquito survival for all the experiments. Results are the mean of three independent experiments (20 mosquitoes/group), and bars represent standard errors of the mean. (0.18 MB TIF) [file pone.0003296.s001.tif]

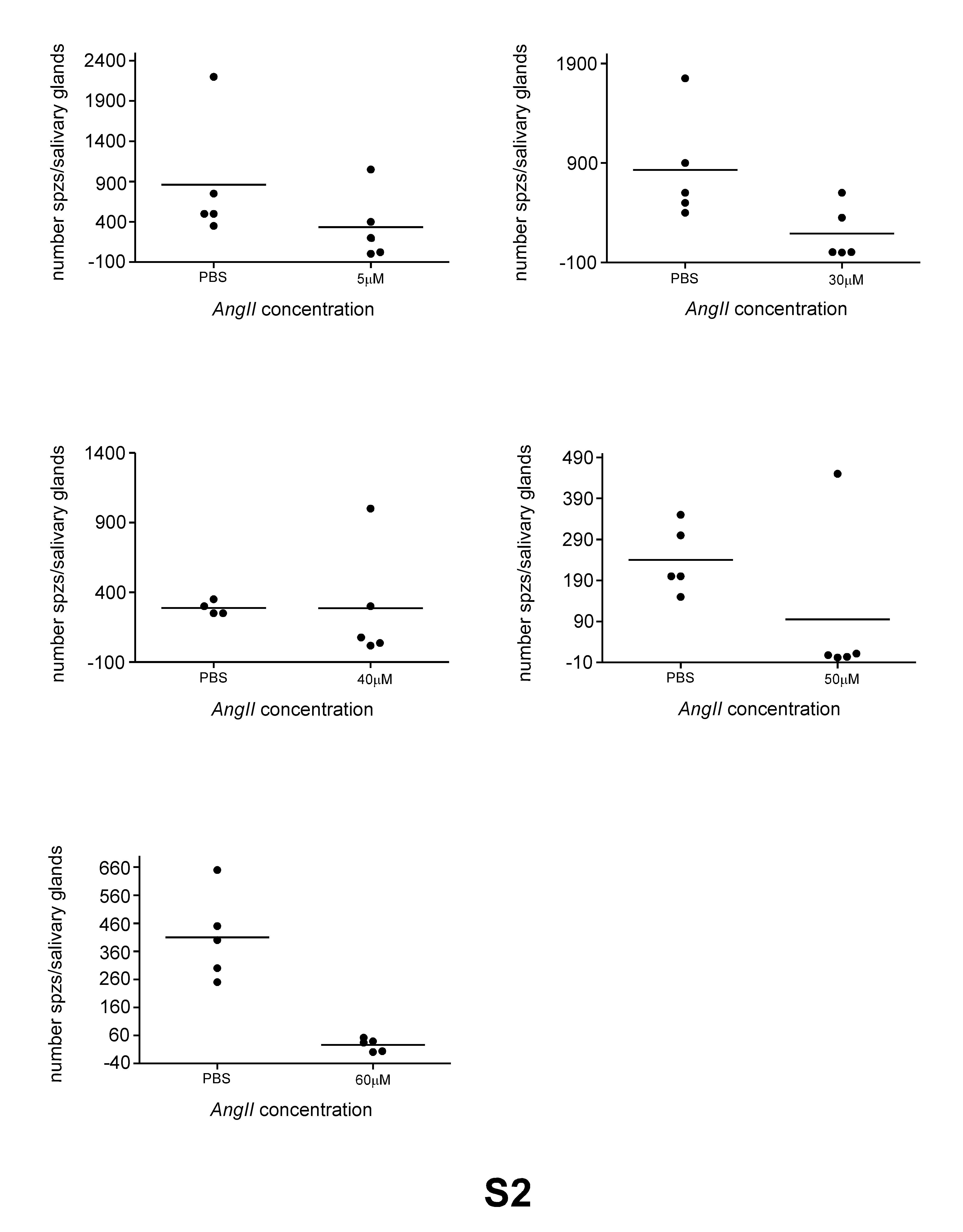

Supplement: Figure S2 — Numbers of sporozoites in salivary glands of angiotensin I-, angiotensin II- or synthetic peptide-treated Ae. aegypti. At day 7 post-infection 0.5 μl of ang II at 5 μM, 30 μM, 40 μM, 50 μM, 60 μM or PBS (control) were injected intrathoracically in anesthetized Ae.aegypti. Salivary glands were dissected 24 hours after the microinjection and sporozoites counted. Mann-Whitney tests indicated significant effects (p<0.0001) of the peptides in the number of salivary glands sporozoites at 60 μM concentration. (0.48 MB TIF) [file pone.0003296.s002.tif]
